# Psychometric validation of the Cardiac Rehabilitation Barriers Scale

**DOI:** 10.1177/0269215511410579

**Published:** 2012-02

**Authors:** Shamila Shanmugasegaram, Lucia Gagliese, Paul Oh, Donna E Stewart, Stephanie J Brister, Victoria Chan, Sherry L Grace

**Affiliations:** 1 York University, Toronto, Ontario, Canada; 2 University Health Network, Toronto General Hospital, Toronto, Ontario, Canada; 3 University of Toronto, Faculty of Medicine, Medical Sciences Building, Toronto, Ontario, Canada; 4 Toronto Rehabilitation Institute, Toronto, Ontario, Canada; 5 York Central Hospital, Ontario, Canada

**Keywords:** Barriers to rehabilitation, cardiac rehabilitation, factor analysis, measurement instrument, psychometry

## Abstract

**Objective:**

The purpose of this study was to investigate the factor structure and psychometric properties of the Cardiac Rehabilitation Barriers Scale (CRBS).

**Design, setting, and participants:**

In total, 2636 cardiac inpatients from 11 hospitals completed a survey. One year later, participants completed a follow-up survey, which included the CRBS. A subsample of patients also completed a third survey which included the CRBS, the Cardiac Rehabilitation Enrolment Obstacles scale, and the Beliefs About Cardiac Rehabilitation scale three weeks later. The CRBS asked participants to rate 21 cardiac rehabilitation barriers on a five-point Likert scale regardless of cardiac rehabilitation referral or enrolment.

**Results:**

Maximum likelihood factor analysis with oblique rotation resulted in a four-factor solution: perceived need/healthcare factors (eigenvalue = 6.13, Cronbach’s *α* = .89), logistical factors (eigenvalue = 5.83, Cronbach’s *α* = .88), work/time conflicts (eigenvalue = 3.78, Cronbach’s *α* = .71), and comorbidities/functional status (eigenvalue = 4.85, Cronbach’s *α* = .83). Mean total perceived barriers were significantly greater among non-enrollees than cardiac rehabilitation enrollees (*P* < .001). Convergent validity with the Beliefs About Cardiac Rehabilitation and Cardiac Rehabilitation Enrolment Obstacles scales was also demonstrated. Test-retest reliability of the CRBS was acceptable (intraclass correlation coefficient = .64).

**Conclusion:**

The CRBS consists of four subscales and has sound psychometric properties. The extent to which identified barriers can be addressed to facilitate greater cardiac rehabilitation utilization warrants future study.

## Introduction

Cardiovascular disease is the leading cause of mortality worldwide^1^ and is a significant contributor to morbidity and health-related costs.^2^ Cardiac rehabilitation, a multidisciplinary approach to secondary prevention, effectively reduces cardiac risk, significantly decreases recurrence of cardiac events, and decreases mortality by 25%.^3^ Despite these well-established benefits of cardiac rehabilitation, it is greatly underutilized, with rates of enrolment ranging from 7.5%^4^ to 29%^5^ and reports of drop-out rates ranging from 40% to 55%.^6–9^ Patient, provider, and health system-level barriers to cardiac rehabilitation utilization have been identified in the literature.^10^

To date, there are psychometrically-validated scales published in the literature assessing cardiac rehabilitation beliefs,^11^ cardiac rehabilitation preferences,^12^ and cardiac rehabilitation enrolment obstacles.^13^ Except for our pilot work,^14–16^ there is no validated multi-level measure of cardiac rehabilitation barriers applicable to both enrolment and participation in cardiac rehabilitation. Firstly, while the Beliefs About Cardiac Rehabilitation scale incorporates some barriers to cardiac rehabilitation utilization, it is only applicable to enrolment but not participation. Secondly, the Cardiac Rehabilitation Preference Form-Revised assesses a patient’s perception of the importance of the cardiac rehabilitation programme features which may be relevant to the degree of cardiac rehabilitation participation, but is not a barrier scale per se. Finally, the Cardiac Rehabilitation Enrolment Obstacles scale was psychometrically-validated in a sample of percutaneous coronary intervention patients only, and therefore its generalizability to other cardiac patients is unknown. Additionally, its criterion validity is weak, as the patient-related obstacles subscale did not differentiate between cardiac rehabilitation enrollees and non-enrollees (*P* = .13).

Grace *et al*. have developed the Cardiac Rehabilitation Barriers Scale (CRBS) which assesses patients’ perceptions of patient, provider and health system-level barriers to cardiac rehabilitation utilization. The scale was developed following an extensive review of the literature, with feedback from cardiologists and cardiac rehabilitation staff. It has been administered to two cardiac cohorts. In the first cohort, researchers administered a 19-item version of the scale to 272 cardiac inpatients from two hospitals.^17^ In the second cohort, investigators administered the same 19-item version of the scale to 1497 cardiac outpatients of 97 cardiologists.^15,16^ The scale discriminated between those who attended cardiac rehabilitation and those who did not, thus illustrating the criterion validity of the scale.^15^ Moreover, analyses revealed differences in cardiac rehabilitation barriers by sex^15^ and age^16^ as have been demonstrated in the literature, thus showing the discriminant ability of the scale.^18–20^ In this study, participants were asked to list additional cardiac rehabilitation barriers in open-ended fashion. Based on these responses, some CRBS items were revised.

The objective of the present study was to psychometrically-validate the revised 21-item version of the CRBS. The psychometric properties to be tested were as follows: (1) factor structure through factor analysis, (2) internal consistency of identified factors, (3) criterion validity with regard to cardiac rehabilitation enrolment and participation, (4) convergent validity with the practical barriers subscale of an adapted version of the Beliefs About Cardiac Rehabilitation^11^ scale and the Cardiac Rehabilitation Enrolment Obstacles^13^ scale, and (5) test-retest reliability.

## Method

As part of a larger study comparing cardiac rehabilitation enrolment following different means of referral, 2636 cardiac inpatients from 11 hospitals between Windsor, Sudbury and Ottawa, Ontario were recruited.^21^ Cardiac rehabilitation services were provided through provincial healthcare at no cost to patients. Ethics approval was granted from all participating institutions. After obtaining consent, clinical data were extracted from medical charts, and a self-report survey was provided to patients for completion. Among other variables, this survey assessed sociodemographic characteristics. One year later, participants were mailed a follow-up survey assessing cardiac rehabilitation utilization and perceived barriers.

The CRBS was re-administered to a subsample of 200 participants from two sites three weeks after they completed the one-year follow-up survey. One institution was a large teaching hospital and the other a small community hospital. The survey also included the Cardiac Rehabilitation Enrolment Obstacles^13^ and the Beliefs About Cardiac Rehabilitation^11^ scales, to establish convergent validity of the CRBS. Thus, most of the study was cross-sectional in design, except for the test-retest assessment.

## Participants

**Figure 1.**
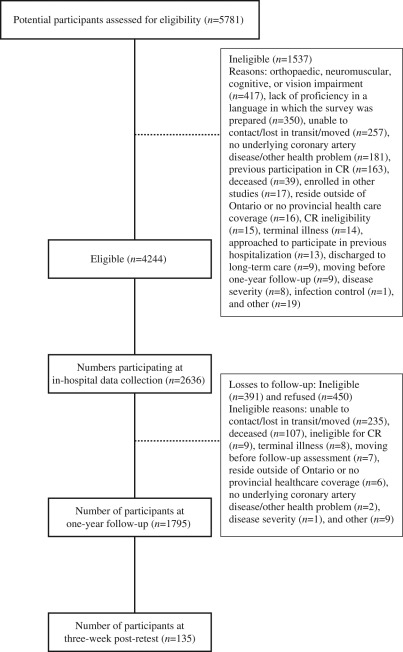
Flow chart of patient recruitment.

Participants consisted of cardiac inpatients. The inclusion criteria at baseline were the following: confirmed acute coronary syndrome diagnosis, patients who had undergone percutaneous coronary intervention or coronary artery bypass graft surgery, and eligibility for cardiac rehabilitation based on indicated cardiac condition.^22^ The exclusion criteria at baseline for the larger study were the following: participation in cardiac rehabilitation within the past two years, and significant orthopaedic, neuromuscular, visual, cognitive or non-dysphoric psychiatric condition which would preclude cardiac rehabilitation participation. The exclusion criteria at one-year follow-up were the following: unable to contact patient, too ill to participate, or deceased. Figure 1 displays the flow diagram of patient participation. The response rate was 62.1% (2636/4244).

## Measures

Self-reported sociodemographic variables measured at baseline through forced-choice options included patient’s marital status, education level, ethnocultural background, family income and work status. Patients were asked at the time of recruitment whether they lived within a 30-minute drive of a hospital, and were coded as rural if they responded ‘no’. Sociodemographic data obtained from the medical chart included date of birth and sex. Clinical variables obtained from the medical chart included blood pressure, lipids, disease severity indicators, and cardiac diagnosis. Key study measures were administered in the follow-up survey mailed one year later. These measures are outlined below.

## The Cardiac Rehabilitation Barriers Scale

The CRBS assesses patients’ perceptions of the degree to which patient, provider, and health system-level barriers affect their cardiac rehabilitation enrolment and participation. Based on pilot-testing, scale revisions resulted in the current 21-item version. Regardless of cardiac rehabilitation referral or enrolment, participants were asked to rate their level of agreement with the statements. Items were rated on a five-point Likert-type scale that ranges from 1 = strongly disagree to 5 = strongly agree. A sample patient-level item is ‘I find exercise tiring or painful’; a sample provider-level item is ‘My doctor did not feel it was necessary’; and a sample health system-level item is ‘I think I was referred, but the rehab program didn’t contact me’. Higher scores indicate greater barriers to patient enrolment or participation in a cardiac rehabilitation programme. Where participants completed more than 80% of the items, a mean score was computed to reflect total cardiac rehabilitation barriers. Where more than 20% of the items were missing, the data were excluded from further analysis.

## Measures to assess the psychometric properties of the CRBS

To investigate the criterion validity of the CRBS, the one-year follow-up survey also assessed self-reported cardiac rehabilitation utilization, through forced-choice response options for enrolment (yes/no), as well as a patient’s estimate of percentage of prescribed cardiac rehabilitation sessions attended.

The Cardiac Rehabilitation Enrolment Obstacles scale^13^ and the Beliefs About Cardiac Rehabilitation scale^11^ were administered in the retest mailing three weeks following the one-year post-recruitment survey. The Beliefs About Cardiac Rehabilitation scale is a 13-item self-report questionnaire, which asks patients to rate their beliefs about cardiac rehabilitation, on a scale from 1 = strongly disagree to 5 = strongly agree. The scale was validated in a sample of 125 acute myocardial infarction (MI) patients at a hospital in the United Kingdom.^11^ Principal components analysis of the original 26 items resulted in a four-factor solution: ‘perceived necessity’, ‘concerns about exercise’, ‘perceived suitability’ and ‘practical barriers’. The Beliefs About Cardiac Rehabilitation scale was intended for individuals who have not yet participated in cardiac rehabilitation. Therefore, in the current study we modified the three items from arguably the most relevant ‘practical barriers’ subscale, so that it would be applicable to both cardiac rehabilitation enrollees and non-enrollees. In particular, the items were phrased in the past tense, such that a sample modified item was: ‘Availability of transport influenced my decision to attend cardiac rehabilitation’.

The Cardiac Rehabilitation Enrolment Obstacles scale is a 15-item self-report questionnaire, which asks patients to indicate the degree to which each item reflects a barrier to enrolment, on a scale from 1 = strongly agree to 5 = strongly disagree. All of the items in this scale are reverse-scored. Researchers examined the preliminary psychometric properties of this scale in a sample of 76 post-percutaneous coronary intervention patients in Australia.^13^ Principal components analysis resulted in a two-factor solution: ‘patient-related obstacles’ and ‘health service-related obstacles’. The researchers also reported that the scale has good internal consistency and satisfactory divergent validity.

## Statistical analyses

SPSS Version 17.0^23^ was used for entering, cleaning, screening and analyzing the data. One-way analysis of variance and chi-square tests were performed to assess differences in in-hospital characteristics among retained, ineligible and declining patients at one-year follow-up and also between participants at one-year follow-up and three-week post-test. Maximum likelihood factor analysis with oblique rotation was used to identify subscales within the CRBS. Factors with eigenvalues greater than one were extracted according to the Kaiser-Guttman criterion.^24^ The scree plot was also used to determine the appropriate number of factors. Factor loadings were interpreted based on loadings of greater than .20 on only one factor.^25^ If an item loaded on multiple factors, then the factor with the highest loading was considered. The internal consistency of the subscales was tested with Cronbach’s alpha.

To assess the criterion validity of the CRBS, independent samples *t*-tests were used to assess differences in mean total scale and subscale scores between cardiac rehabilitation enrollees and non-enrollees, and Pearson’s correlation analysis was used to assess the degree of association with self-reported percentage of prescribed cardiac rehabilitation sessions attended. The convergent validity was assessed by examining the degree of association among the mean total CRBS and its subscales, the Beliefs About Cardiac Rehabilitation practical barriers subscale and the Cardiac Rehabilitation Enrolment Obstacles scale through Pearson’s correlation analysis.

Intraclass correlation analysis using two-way mixed average measures was performed to determine the three-week test-retest reliability of the CRBS. Specifically, the association between the mean total CRBS score on the one-year follow-up survey was compared with the CRBS score for the survey administered three weeks later.

## Results

Table 1 displays the in-hospital characteristics of retained, ineligible, and declining patients at one-year follow-up. The retention rate was 80.0%. Compared to ineligible and declining patients, retained participants were significantly more likely to be married and/or have a current or previous history of coronary artery bypass graft surgery. In addition, the retained participants were less likely to smoke and/or have a current or previous history of MI compared to ineligible and declining patients.

**Table 1.** Baseline characteristics of retained, ineligible, and declining patients at one-year follow-up (*n* = 2636)

Characteristic	Retained participants (*n* = 1795)	Ineligibles (*n* = 391)	Declined (*n* = 450)	Total (*n* = 2636)	Three-week post-test participants (*n* = 135)
**Sociodemographic**					
Age (mean ± SD)	65.4 ± 10.4	66.6 ± 13.0	61.8 ± 12.2^‡‡‡^	65.0 ± 11.2	64.9 ± 10.5
Sex (% female)	447 (24.9)	130 (33.3)^††^	130 (28.9)	707 (26.8)	45 (33.3)^§^
Marital status* (% married)	1380 (77.7)	239 (62.1)^†††^	297 (67.5)^†††^	1916 (73.7)	102 (75.6)^§§§^
Ethnocultural background* (% minority)	284 (16.5)	69 (19.0)	108 (25.9)^†††‡^	461 (18.4)	28 (21.5)^§§§^
Education* (% >high school)	844 (48.5)	140 (37.6)^†††^	224 (51.6)^‡‡‡^	1208 (47.5)	77 (57.5)^§^
Family income* (% >$50,000 CAD)	722 (49.8)	101 (34.2)^†††^	166 (47.7)^‡‡^	989 (47.3)	56 (49.1)
Work status* (% full or part-time)	618 (35.8)	102 (27.5)^††^	193 (46.2)^†††‡‡‡^	913 (36.3)	43 (33.6)
Rurality* (% yes)	214 (11.9)	59 (15.2)	52 (11.7)	325 (12.4)	11 (8.1)
**Clinical**					
Systolic BP mm Hg (mean ± SD)	127.7 ± 19.0	127.2 ± 21.5	129.6 ± 22.9	128.0 ± 20.0	128.6 ± 18.5
Diastolic BP mm Hg (mean ± SD)	71.1 ± 11.5	70.2 ± 12.6	72.8 ± 13.9^‡^	71.3 ± 12.1	72.6 ± 11.9
Total Cholesterol/HDL ratio (mean ± SD)	4.37 ± 2.69	4.24 ± 1.44	4.52 ± 1.79	4.38 ± 2.43	4.46 ± 1.76
HDL mmol/L (mean ± SD)	1.06 ± .39	1.01 ± .32	1.04 ± .40	1.05 ± .38	1.22 ± .56^§§§^
LDL mmol/L (mean ± SD)	2.47 ± 1.04	2.36 ± .95	2.47 ± 1.12	2.46 ± 1.04	2.65 ± 1.17
NYHA Class II-IV (%)	116 (26.7)	37 (49.3)^†††^	27 (22.5)^‡‡‡^	180 (28.6)	6 (9.5)^§§^
CCS angina class 2-4 (%)	534 (83.7)	85 (87.6)	124 (82.1)	743 (83.9)	58 (80.6)
Duke Activity Status Index* (mean ± SD)	27.9 ± 17.2	21.9 ± 17.5^†††^	27.5 ± 18.7^‡‡‡^	26.9 ± 17.6	34.0 ± 15.6^§§§^
BMI* (mean ± SD)	28.2 ± 5.3	28.1 ± 6.3	29.0 ± 6.0^†^	28.4 ± 5.6	28.9 ± 4.28
Smoking status* (% current)	111 (6.4)	37 (10.1)^†^	56 (13.2)^†††^	204 (8.1)	9 (7.0)
Current or Previous MI (% yes)	499 (28.0)	127 (33.4)^†^	145 (32.9)^†^	771 (29.6)	12 (8.9)^§§§^
Current or Previous PCI (% yes)	595 (33.4)	94 (24.8)^††^	170 (38.5)^†‡‡‡^	859 (33.0)	99 (73.3)^§§§^
Current or Previous CABG (% yes)	738 (41.4)	100 (26.4)^†††^	128 (29.0)^†††^	966 (37.1)	9 (6.7)^§§§^
Current or Previous HF (% yes)	175 (9.8)	92 (24.3)^†††^	57 (12.9)^‡‡‡^	324 (12.4)	6 (4.4)^§^
Current or Previous Arrhythmia (% yes)	223 (12.5)	50 (13.2)	50 (11.3)	323 (12.4)	11 (8.1)
Current or Previous Valve repair (% yes)	40 (28.2)	3 (14.3)	4 (22.2)	47 (26.0)	2 (66.7)

Note: Percentages take into account missing data for some variables.

BMI, body mass index; BP, blood pressure; CABG, coronary artery bypass graft; CAD, Canadian dollar; CCS, Canadian Cardiovascular Society; HF, heart failure; HDL, high-density lipoprotein; LDL, low-density lipoprotein; MI, Myocardial Infarction; NYHA, New York Heart Association; PCI, percutaneous coronary intervention; SD, standard deviation.

* Presents self-report data. All other data elements extracted from patient charts.

Significant difference between participants and ineligibles (denoted only in ineligibles’ column) ^†^*P* < .05; ^††^*P* < .01; ^†††^*P* < .001. Significant difference between participants and decliners (denoted only in decliners’ column) ^†^*P* < .05; ^††^*P* < .01; ^†††^*P* < .001.

Significant difference between ineligibles and decliners (denoted only in decliners’ column) ^‡^*P* < .05; ^‡‡^*P* < .01; ^‡‡‡^*P* < .001.

Significant difference between retained participants at one-year follow-up and three-week post-test (denoted only in three-week post-test participants’ column) ^§^*P* < .05; ^§§^*P* < .01; ^§§§^*P* < .001.

The table also presents the sociodemographic and clinical characteristics of the subsample of participants who completed the final survey. Compared to those who completed the one-year survey, the three-week post-test survey sample consisted of significantly more females. In addition, these participants were significantly more likely to belong to an ethnic minority group, have an education level greater than high school, greater high-density lipoprotein (HDL) level, report greater activity status, and have a current or previous history of percutaneous coronary intervention compared to one-year follow-up participants. Moreover, the three-week post-test participants were significantly less likely to be married, have New York Heart Association functional class between II and IV, current or previous history of MI, coronary artery bypass graft surgery, and/or heart failure.

Participants with less than five per cent of the CRBS scale items missing (i.e. individuals with a maximum of one item missing) were selected for further analyses. Missing Value Analysis^26^ was performed on these participants, and results revealed that the data were not missing completely at random. Thus, the Expectation-Maximization method was used for estimating statistics. The data imputation file was merged with the original file prior to further analyses. Finally, the mean for the total CRBS, the mean for the Beliefs About Cardiac Rehabilitation practical barriers subscale, and the sum for the Cardiac Rehabilitation Enrolment Obstacles subscales were computed only for participants with less than 20% of the scale items missing. Data were screened for normality and linearity of variables, and outliers prior to analysis.

### Factor structure of the CRBS

The initial stage of factor analysis included screening for multicollinearity and singularity. Maximum likelihood factor analysis with oblique rotation was then performed on the 21 items from the CRBS. Loading of variables on factors, percentage of variance, eigenvalues, and internal consistency of each subscale and means and standard deviations of the items are shown in Table 2.

**Table 2.** Maximum likelihood factor analysis, percentage of variance, eigenvalues, and reliability of each factor (*n* = 958)

CRBS item	Health Care	Logistical	Work/Time	Comorbidities	*Mean*	*SD*
…I don’t need CR	**.85**	−.01	.01	−.11	2.02	1.20
…I can manage on my own	**.83**	.08	.06	.00	1.93	1.05
…my doctor didn’t feel it was necessary	**.77**	.09	−.04	.13	1.91	1.08
…many people with heart problems don’t go to CR and they are fine	**.67**	.04	.06	.22	1.86	.99
…I prefer to take care of my health alone	**.57**	−.08	.13	.10	2.00	1.12
…I already exercise at home or in my community	**.57**	−.22	.05	−.19	2.59	1.39
…I didn’t know about CR	**.54**	−.15	−.11	.12	1.96	1.30
…I think I was referred but the rehab program didn’t contact me	**.34**	−.13	.05	.30	1.73	.97
…it took too long to get referred and into the program	**.32**	−.18	.07	.22	1.86	1.03
…of cost	.01	−**.85**	−.03	−.01	2.05	1.26
…of transportation problems	−.02	−**.84**	−.04	.11	1.90	1.12
…of distance	.06	−**.83**	.01	−.08	2.16	1.35
…of family responsibilities	.03	−**.57**	.18	.05	1.90	1.09
…severe weather	−.04	−**.50**	.12	.13	2.08	1.22
…of work responsibilities	.04	.04	**.87**	−.07	2.13	1.23
…of time constraints	.09	−.06	**.75**	.00	2.08	1.18
…travel	−.07	−.05	**.36**	.13	2.22	1.25
…I don’t have the energy	.04	−.07	.08	**.73**	1.97	1.12
…I find exercise tiring or painful	.06	−.15	−.03	**.63**	2.01	1.19
…other health problems prevent me from going	.00	.00	.09	**.61**	2.07	1.17
…I am too old	.31	−.06	.05	**.55**	1.65	.84
Variance explained	38.66%	7.81%	4.86%	4.06%		
Eigenvalue	6.13	5.83	3.78	4.85		
Reliability	.89	.88	.71	.83		

CR, cardiac rehabilitation; SD, standard deviation.

Reproduced with kind permission from Cardiac Rehabilitation Barriers Scale, 21-Items, ©CRBS-21.

Four factors were extracted. All factors were internally consistent and well-defined by the items. The first factor reflects perceived need/healthcare factors. The second factor reflects logistical factors such as distance and cost. The third factor reflects work/time conflicts. The fourth factor reflects comorbidities/functional status.

### Criterion validity

Nine hundred and forty-eight (54.7%) patients self-reported enrolling in cardiac rehabilitation at one of 60 sites, and attended 82.7±27.3% of prescribed sessions. Table 3 displays mean total and subscale CRBS scores for cardiac rehabilitation enrollees and non-enrollees at one-year follow-up. Mean total cardiac rehabilitation barriers were significantly greater among non-enrollees than enrollees. In addition, non-enrollees had significantly greater scores on the healthcare, logistical factors and comorbidities/functional status subscales than enrollees. Enrolment was significantly related to the mean total CRBS (*P* < .001) and Beliefs About Cardiac Rehabilitation scale (*P* < .05), but not to the total Cardiac Rehabilitation Enrolment Obstacles scale (*P*>.05) in the three-week post-test subsample. Mean total CRBS (*r* = −.35, *P* < .001), healthcare factors (*r* = −.36, *P* < .001), logistical factors (*r* = −.26, *P* < .001), work/time conflicts (*r* = −.18, *P* < .001), and comorbidities/functional status (*r* = −.33, *P* < .001) subscale scores were significantly and negatively related to percentage of cardiac rehabilitation session attendance at one-year follow-up. For the three-week post-test subsample, degree of participation was significantly related to the mean total CRBS (*r* = −.50, *P* < .01), but not to the total Cardiac Rehabilitation Enrolment Obstacles scale (*r* = −.05, *P* > .05) or mean total Beliefs About Cardiac Rehabilitation scale (*r* = −.04, *P* > .05).

**Table 3.** Criterion validity of the CRBS (*n* = 1763)

	Enrollees (*n* = 950)		Non-enrollees (*n* = 813)		
Mean total/subscale	*Mean*	*SD*	*Mean*	*SD*	*t*
Mean total CR barriers	1.77	.66	2.46	.61	−17.09*
Healthcare	1.64	.70	2.67	.65	−25.21*
Logistical	1.86	.95	2.45	1.03	−9.32*
Work/time conflicts	2.19	1.04	2.22	.95	−.58
Comorbidities/functional status	1.78	.86	2.35	.96	−10.44*

* *P* < .001.

CR, cardiac rehabilitation; SD, standard deviation.

### Convergent validity and test-retest reliability

Table 4 displays the correlation coefficients for the relationships among mean total and subscale scores of the CRBS, with the Cardiac Rehabilitation Enrolment Obstacles subscales and the practical barriers subscale of the Beliefs About Cardiac Rehabilitation scale. Mean total CRBS and the healthcare factors subscale of the CRBS were significantly and positively related to the health service-related obstacles subscale of the Cardiac Rehabilitation Enrolment Obstacles scale, and the work/time conflicts subscale was significantly and positively related to the patient-related obstacles subscale of the Cardiac Rehabilitation Enrolment Obstacles scale. In addition, mean total CRBS and all four subscales were significantly and positively related to the practical barriers subscale of the Beliefs About Cardiac Rehabilitation scale.

**Table 4.** Convergent validity of the CRBS (*n* = 135)

Subscale	Mean total	Healthcare	Logistical	Work/time	Comorbidities
CREO Patient	.18	.07	.14	.30*	.12
CREO Health	.26*	.38**	.12	.01	.09
BACR Practical	.53**	.32**	.57**	.33**	.36**

* *P* < .01; **P < .001.

BACR, Beliefs About Cardiac Rehabilitation scale; CREO, Cardiac Rehabilitation Enrolment Obstacles scale.

There was a 68% response rate for the re-test administration of the CRBS (135/200). Test-retest reliability of the CRBS was acceptable (intraclass correlation coefficient = .64, *P* < .001).

## Discussion

This study sought to validate a multi-level cardiac rehabilitation barriers scale, applicable to both enrollees and non-enrollees alike. Factor analysis revealed four subscales, namely perceived need/healthcare factors, logistical factors, work/time conflicts, and comorbidities/functional status. All of the subscales had good internal consistency. CRBS scores were significantly related to enrolment status and degree of cardiac rehabilitation participation, such that the criterion validity of the CRBS was established. Finally, convergent validity was demonstrated, and test-retest reliability was acceptable.

Based on evidence from almost 50 trials and approximately 9000 patients,^3^ many clinical practice guidelines promote cardiac rehabilitation as a standard part of the continuum of care.^10^ Unfortunately, there is gross underutilization of these services, and wide variation by region and population.^9,27^ While many barriers to cardiac rehabilitation utilization have been identified in the literature, to date there have been limited means to assess these in a robust manner. Overall, the CRBS may enable identification of individuals who face barriers to cardiac rehabilitation utilization. Ultimately, these barriers may be amenable to modification or intervention, thus potentially increasing cardiac rehabilitation utilization and facilitating optimal patient recovery and outcomes.

### Factor structure of the CRBS

Results of the present study are generally consistent with those reported previously.^17^ In particular, the logistics and work/time conflicts subscales were highly concordant. However, there are two differences in these studies. Firstly, the present study revealed a fourth factor – comorbidities/functional status. This factor consisted of two items that were added to this version of the scale based on open-ended responses from participants in the previous cohort. The two new items were: ‘I don’t have the energy’ and ‘I am too old’. Secondly, items that were categorized as ‘denial/minimization of heart disease’ were rephrased and incorporated as ‘perceived need/healthcare factors’ in the current loading matrix. This discrepancy is chiefly a function of semantics. Indeed, the items loading on this factor from both scale versions are highly concordant. This factor includes items such as ‘I don’t need cardiac rehabilitation’, and ‘I didn’t know about cardiac rehabilitation’. These variables appear to relate to patients’ perceived need for cardiac rehabilitation as a result of both provider and health system-level issues, which mitigate cardiac rehabilitation utilization. Research has shown that strength of physician endorsement is a key factor in whether patients enroll in cardiac rehabilitation programmes.^28^ In addition, research has established that barriers at the level of the health system, notably type of cardiac rehabilitation referral, are paramount. Thus, provider and health system-level barriers could contribute to patients’ lack of knowledge about the benefits of cardiac rehabilitation, and result in patients’ minimization of their need for cardiac rehabilitation. Although these results are consistent with the results reported by Grace *et al*. (2004), future research is needed to conduct confirmatory factor analysis using structural equation modelling perhaps to replicate the current investigation.

### Other psychometric characteristics of the CRBS

Overall, these results support the criterion validity of the CRBS. Findings suggest that both healthcare and patient factors are important in cardiac rehabilitation enrolment decisions as well as degree of cardiac rehabilitation participation. It is here that the psychometric characteristics of the CRBS may be superior to the Cardiac Rehabilitation Enrolment Obstacles, in that the patient-related obstacles subscales of the Cardiac Rehabilitation Enrolment Obstacles scale did not discriminate between enrollees and non-enrollees.^12^ The Cardiac Rehabilitation Enrolment Obstacles and Beliefs About Cardiac Rehabilitation scales serve somewhat different purposes than the CRBS, so researchers and potentially clinicians should choose the scale that best serves their specific needs.

In terms of convergent validity, results suggest that the CRBS indeed measures barriers at multiple levels. Finally, three-week test-retest reliability was acceptable. This suggests that the measure is stable. However, it would be of interest to assess cardiac rehabilitation barriers across time to see whether they change; for example, from the point of diagnosis, through intervention and disease progression.

### Implications

While the CRBS has been developed for research purposes, future research is needed to investigate whether it would also be useful for policy and clinical purposes. The CRBS could facilitate identification of the most significant barriers across regions, and also particular to certain models of healthcare organization. These barriers may ultimately be amenable to modification or intervention, thus increasing cardiac rehabilitation utilization and potentially optimizing patient outcomes. For instance, implementation of automatic referral to cardiac rehabilitation, along with a healthcare provider informing patients about its benefits, could help patients overcome the most-endorsed barriers which were ‘I already exercise at home or in my community’, ‘travel’ and ‘distance’ in the current sample. However, the retrospective nature of the design limits interpretability of the findings at this stage.

Provider acceptability, time to complete during busy medical appointments, and determination of the effectiveness of interventions to address identified barriers would need to be established to ascertain clinical utility. The CRBS might be administered to inpatients or outpatients during specialist or primary care appointments. It may even be useful at a cardiac rehabilitation intake appointment to identify any barriers to full programme participation. This tool may enable screening and detection of individuals who face barriers to both cardiac rehabilitation enrolment and participation.

The limitations of this study chiefly pertain to recall bias. This may be at play as a result of the amount of time that would have elapsed between healthcare provider interactions where cardiac rehabilitation may have been discussed, and completion of the one-year follow-up survey when the CRBS was administered. It is unknown at what point patients may have experienced or developed cardiac rehabilitation barriers. In addition, the CRBS was administered one-year post-hospitalization and may not be reflective of barriers at other time points along the continuum of care or disease. Future research is needed to explore the predictive validity of the CRBS. Third, patient-report of perceived healthcare provider and health system-level cardiac rehabilitation barriers may be erroneous. Fourth, the generalizability of findings may be hindered for several reasons. There was some bias in the characteristics of the retained sample and the three-week post-test subsample. Moreover, this study was conducted in Ontario, which has a universal healthcare coverage system for cardiac rehabilitation. Thus, these findings are generalizable only to patients in regions that have a similar healthcare coverage system.

In conclusion, while scales of cardiac rehabilitation beliefs and preferences have been developed recently, the CRBS is the first scale to assess multi-level cardiac rehabilitation barriers for both enrollees and non-enrollees. The results presented herein indicate that the CRBS is a reliable and valid measure of cardiac rehabilitation barriers. The availability of the CRBS, among other recently-published cardiac rehabilitation scales, may enable identification of key barriers for individual patients and in subgroups that are underrepresented in cardiac rehabilitation, as well as comparison of barriers across studies, populations, and jurisdictions.

**Clinical messages**

- The Cardiac Rehabilitation Barriers Scale assesses patients’ perceptions of patient, provider and system-level barriers.
- It is a reliable and valid measure and consists of four subscales.
- This comprehensible tool may enable screening and detection of individuals who face barriers to both cardiac rehabilitation enrolment and participation.
